# The Efficacy of Fisheries Management: A Length-Based Stock Assessment of Eight Fish Species in Xingkai Lake, China

**DOI:** 10.3390/ani15223350

**Published:** 2025-11-20

**Authors:** Chen Zhao, Zhongsi Gao, Xuehao Wang, Wanting Wang, Huibo Wang, Le Wang, Tangbin Huo

**Affiliations:** 1 Key Laboratory of Aquatic Organism Protection and Ecological Restoration in Cold Waters, Heilongjiang River Fisheries Research Institute, Chinese Academy of Fishery Sciences, Harbin 150070, China; zhaochen@hrfri.ac.cn (C.Z.); gaozhongsi@hrfri.ac.cn (Z.G.); wxh160566@163.com (X.W.); phtwwt@163.com (W.W.); wanghuibo@hrfri.ac.cn (H.W.); wangle@hrfri.ac.cn (L.W.); 2 College of Fisheries and Life Science, Dalian Ocean University, Dalian 116023, China; 3 College of Life Science and Technology, Mudanjiang Normal University, Mudanjiang 157011, China

**Keywords:** LBB method, overfishing, stock assessment, sustainable fisheries management, fishery resources, Xingkai Lake

## Abstract

Standardized fishing gear represents a common fisheries management intervention, yet its effectiveness in multi-species freshwater ecosystems remains insufficiently assessed. This study presents the first application of the Length-Based Bayesian Biomass (LBB) method to systematically evaluate the stock status of eight major commercial fish species in Xingkai Lake (located within China). Length-frequency data collected in 2019 (marking the policy initiation) and 2024 (after five years of implementation) were used to estimate key population parameters and exploitation indicators. The results revealed that the four species (*Carassius gibelio*, *Acheilognathus macropterus*, *Hemiculter lucidus*, and *Hemiculter leucisculus*) recovered to healthy status (*B/B_MSY_* ≥ 1.0), demonstrating the policy’s effectiveness for small-bodied, fast-growing species. In contrast, three species (*Chanodichthys abramoides*, *Chanodichthys mongolicus*, and *Chanodichthys erythropterus*) remained grossly overfished (*B/B_MSY_* < 0.5), while *Culter alburnus* experienced increased fishing pressure, indicating limited efficacy for larger, slow-growing species with collapsed initial stocks. Although the mesh size increase facilitated recovery of specific populations, fisheries resources in Xingkai Lake continue to face overfishing pressure. Management strategies lacking an ecosystem perspective may not only fail to achieve conservation objectives but could also trigger ecological risks. This study underscores that ecosystem-based management is essential for sustainable management of multi-species fisheries.

## Simple Summary

Overfishing has led to a continuous decline in fishery resources in Xingkai Lake, the largest transboundary freshwater lake in Asia, though the specific status of fish populations has lacked scientific assessment. To promote resource recovery, local authorities implemented a large-mesh fishing gear policy in 2019, yet its effectiveness remained unclear. This study provides the first systematic evaluation of the impact of this five-year policy on the recovery of eight major commercial fish species in the lake. The results show that while increasing mesh size effectively promoted the rapid recovery of small fish species, medium and large species will require more time to rebuild their populations. Overall, the lake’s fisheries remain in an overexploited state. This indicates that a single management measure is unlikely to achieve optimal outcomes for all fish species and may even pose potential risks of unbalancing fish community structure. This research offers key scientific evidence for developing more targeted, multi-species collaborative management strategies, providing important guidance for achieving sustainable fisheries and ecological balance in Xingkai Lake.

## 1. Introduction

Overfishing represents a predominant driver of the global decline in fishery resources, leading to the depletion of numerous fish stocks worldwide [1]. This overexploitation not only diminishes biodiversity but can also compromise ecosystem functioning and induce regime shifts [2]. Compared to marine ecosystems, freshwater systems have received comparatively limited attention in biodiversity conservation and sustainable fishery management [3]. Pressing issues such as environmental pollution, habitat degradation, and overfishing pose serious threats to the health and security of freshwater ecosystems [4]. Unassessed fisheries account for over 80% of the global catch; the stock dynamics and exploitation status in most aquatic systems remain poorly understood [5]. According to the FAO’s Review of the State of World Marine Fishery Resources—2025, more than one-third of global fish stocks are subject to unsustainable fishing pressure, with the rate of overfishing increasing at an average of approximately 1% per year in recent decades [6]. The report further highlights that small-scale and artisanal fisheries dominate in developing countries, where insufficient data and limited management capacity represent major impediments to sustainable development [6]. Therefore, a comprehensive assessment of current exploitation status and resource trends is essential for fisheries management authorities to evaluate sustainable exploitation potential [7].

Conventional stock assessment methods typically depend on extensive catch statistics with age structures and detailed biological surveys, which are often costly and logistically challenging to acquire [8]. Such data limitations hinder the implementation of robust stock assessments and species-specific management strategies in data-poor contexts [9]. In contrast, length-based methods allow for the estimation of population parameters and stock status using more readily available data [10]. In 2018, Froese et al. introduced the Length-based Bayesian Biomass (LBB) method, which estimates key population parameters and exploitation rates based solely on length-frequency data [11]. A principal advantage of the LBB method is its capacity to automatically evaluate data validity, thereby improving the reliability of estimation results [11]. This approach has been widely applied to assess fish stock status, evaluate impacts of different fishing practices, and support sustainable fisheries management strategies [12,13].

Xingkai Lake, the largest transboundary lake in Asia, is shared by China and Russia [14]. The Chinese sector was designated as a National Nature Reserve in 1994, making it the only nature reserve in China protected under a specific bilateral agreement with Russia [15]. It gained further international status as a Ramsar Wetland of International Importance (2002) and a UNESCO World Biosphere Reserve (2007), highlighting its global significance for biodiversity [16]. Despite these protections, the reserve’s unique lake–forest–wetland ecosystem faces severe threats from ecological degradation and overfishing, leading to declining fishery resources [17]. While historical records list 60 indigenous fish species, fewer than 10 now hold commercial value [17]. The fisheries in Xingkai Lake are exclusively small-scale, with an average of one non-mechanized fishing boat per 5 km^2^ [18]. Nevertheless, these operations exert severe pressure on the lake’s fishery resources due to uncontrolled expansion of fishing effort [18]. The management of these fisheries is particularly challenging, posing difficulties despite their smaller crew sizes due to widespread illegal harvesting of juveniles and broodstock, a problem exacerbated in developing regions [19].

Effective management of these fisheries requires comprehensive strategies, including restrictions on fishing practices and gear types, implementation of total allowable catch (TAC), enforcement of minimum and maximum size limits for landed fish, and seasonal or spatial fishing closures [20]. To promote sustainable fisheries, local authorities have implemented an annual fishing moratorium from 6 June to 15 July and designated core reserve areas as no-fishing zones. In 2019, fishing gear was standardized through the prohibition of nets targeting small adult fish and the mandatory use of gillnets with a minimum mesh size of 10 cm. However, the efficacy of these measures remains uncertain due to the absence of systematic evaluation. Fisheries management in Xingkai Lake has historically been constrained by a lack of quantitative assessments, which has limited the scientific foundation for conservation interventions. Therefore, a systematic assessment of the principal commercial fish species in Xingkai Lake is urgently needed. This study addresses this critical gap by conducting the first systematic evaluation of fishery resource utilization in the Xingkai Lake National Nature Reserve. Using length-frequency data collected in 2019 and 2024 for eight major indigenous commercial species, we applied the LBB method to estimate population parameters and assess exploitation status. Our analysis evaluates both the effectiveness and limitations of the 2019 fishing gear standardization policy and provides science-based recommendations to support future management strategies.

## 2. Materials and Methods

### 2.1. Data Sources

**Figure 1:**
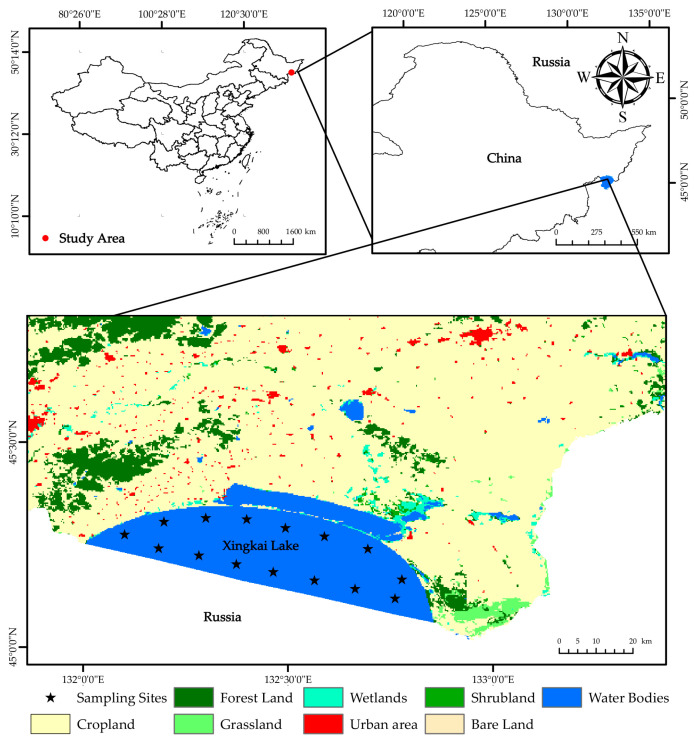
Sampling sites of the Xingkai Lake (located within China).

Biological data were collected monthly during the ice-free months (May to November) in 2019 and 2024 within Xingkai Lake (located within China). Sampling was conducted at 15 sites, approximately 5 km apart, with their locations shown in Figure 1. The land use data in this figure are derived from reference [21]. At each site, two multi-mesh gillnets (100 m long × 3 m high) with mesh sizes of 1.0, 2.0, 3.1, 4.0, 4.8, 7.5, 8.5, 11.0, and 14.0 cm were deployed for 24 h. All captured individuals of the eight species—*Hemiculter leucisculus*, *Hemiculter lucidus*, *Carassius gibelio*, *Acheilognathus macropterus*, *Culter alburnus*, *Chanodichthys mongolicus*, *Chanodichthys abramoides*, and *Chanodichthys erythropterus*—were measured for body length to the nearest 0.1 cm and body weight to the nearest 0.1 g.

### 2.2. Description of the LBB Method

Length-frequency data were constructed from individual length measurements using fixed class intervals determined by the maximum recorded body length (*L_max_*) of each species. The intervals were assigned as follows: 0.3 cm for species with *L_max_* ≤ 12 cm, 0.5 cm for species with 12 cm < *L_max_* ≤ 20 cm, 1.0 cm for species with 20 cm < *L_max_* ≤ 35 cm, and 2.0 cm for the largest species with *L_max_* > 35 cm [13].

The LBB method was applied to analyze these data within a Bayesian framework, assuming that growth in length follows the von Bertalanffy growth function (VBGF) [11]. A Bayesian framework with Markov Chain Monte Carlo (MCMC) sampling estimated all population parameters. As the foundational formulas of the LBB method have been thoroughly described by Froese et al. [11], only the key equations relevant to the model construction process are presented herein.

(1) $$L_{t}={\;L}_{\inf}\left[1-e^{-k\left(t-t_{0}\right)}\right]$$

where *L_t_* represents body length at age *t*, *L_inf_* signifies asymptotic length, *K* is the growth coefficient, and *t*_0_ denotes the theoretical age of the fish at zero length.

The survival process is described by

(2) $$N_{L}=N_{L_{\mathrm{start}}}{\left(\frac{L_{\inf}-L}{L_{\inf}-L_{\mathrm{start}}}\right)}^{Z/K}$$

where *N_L_* corresponds to the number of fish surviving at length *L*, $N_{L_{\mathrm{start}}}$ represents the number at the length *L_start_*, and *Z*/*K* indicates the ratio of total mortality (*Z*) to growth rate (*K*).

Under natural conditions without fishing, the total mortality coefficient (*Z*) is equal to the natural mortality coefficient (*M*). In this case, *L_start_* is set to 0 and $N_{L_{\mathrm{start}}}\;$ to 1. The probability of survival to relative length is given by

(3) $$P_{L/L_{\inf}}={(1-\frac{L}{L_{\inf}})}^{M/K}$$

where $P_{L/L_{\inf}}$ represents the probability of a fish surviving to a relative length *L*/*L_inf_*, a function governed by the *M*/*K* ratio. This implies that any fish species sharing the same *M*/*K* ratio will have an identical probability of reaching its asymptotic length.

The LBB method can incorporate various gear-selection functions. Since fishing gear type significantly influences catch composition, this study applied a Gaussian selection function appropriate for gillnets, as defined by the following equations:

(4) $$S_{L}\;={\;e}^{-\frac{{\left(L-L_{c}\right)}^{2}}{2\sigma^{2}}}$$

where *S_L_* is the selectivity coefficient at length *L*, *σ* determines the standard deviation of the selectivity curve, a parameter that determines its width and steepness, and *L_c_* is the length-at-maximum-selectivity, representing the body length at which the capture probability is highest, corresponding to the peak of the curve.

Equation (2) has to be replaced by a difference equation fitted to the whole catch-in-numbers curve to estimate *L_inf_*, *L_c_*, *σ*, *M*/*K*, and *F*/*K* simultaneously:

(5) $$N_{L_{i}}\;=\;N_{L_{i-1}}{\left(\frac{L_{\inf}-L_{i}}{L_{\inf}-L_{i-1}}\right)}^{\frac{M}{K}+\frac{F}{K}S_{L_{i}}}$$

where $N_{L_{i}}$ is the number of individuals in the length group *L_i_*, and $N_{L_{i-1}}$ denotes the number of individuals in the previous length group.

The optimal length for maximum cohort biomass (*L_opt_*) was derived as

(6) $$L_{\mathrm{opt}}=L_{\inf}\left(\frac{3}{3+\frac{M}{K}}\right)$$

The corresponding optimal length at first capture (*L_c_opt_*) was calculated using

(7) $$L_{c\text{\_}\mathrm{opt}}=\frac{L_{\inf}\left(2+3\frac{F}{M}\right)}{\left(1+\frac{F}{M}\right)\left(3+\frac{M}{K}\right)}$$

Since CPUE is proportional to the biomass of the stock during the exploitation phase, the ratio of the exploitable fraction (>*L_c_*) of unexploited biomass (*B*_0_) to the catch per unit of effort index (*CPUE*′/*R*) yields a relative biomass index for the exploited fraction (*B*/*B*_0_):

(8) $$\frac{B}{B_{0}}\;=\;\frac{\frac{{\mathrm{CPUE}}^{\prime }}{R}}{\frac{{B_{0}}^{\prime }\;>\;L_{c}}{R}}$$

The analysis was implemented with the Bayesian Gibbs sampler software JAGS (Just Another Gibbs Sampler) version 4.3.0, a program designed for Markov Chain Monte Carlo (MCMC) simulation of Bayesian hierarchical models. The length-frequency (LF) data were processed using the R statistical language (version 4.1.3) and the dedicated R script (LBB_33a.R) developed by Froese et al. [11]. This script is publicly available and can be downloaded from the open-access repository at http://oceanrep.geomar.de/44832/ (accessed on 15 October 2024).

In addition, we calculated the exploitation rate (*E*), which enables the observation of over (when >0.5) or under (0.5) exploitation, with the equation *E* = *F*/*Z* [13].

### 2.3. Assessment Criteria

The assessment of fishery stocks utilized multiple reference points to evaluate population status [11]. Based on the *B*/*B_MSY_* ratio, stocks were classified into five distinct categories: healthy status (*B*/*B_MSY_* ≥ 1.0), slightly overfished status (0.8 ≤ *B*/*B_MSY_* < 1.0), overfished status (0.5 ≤ *B*/*B_MSY_* < 0.8), grossly overfished status (0.2 ≤ *B*/*B_MSY_* < 0.5), and collapsed (*B*/*B_MSY_* < 0.2) [11,22].

Additional indicators provided complementary insights into stock condition. The fishing mortality to natural mortality ratio (*F*/*M*) assessed exploitation pressure, with *F*/*M* > 1.0 confirming overfishing [23]. Relative biomass (*B*/*B*_0_) below 0.5 reflected severe stock depletion [24]. Length-based indicators revealed structural changes in populations: *L_mean_*/*L_opt_* and *L_c_*/*L_c_opt_* ratios below unity indicated truncated size structure and capture of undersized individuals, while *L_95th_*/*L_inf_* approaching or exceeding 0.9 suggested at least some large fish were still present [24].

These integrated criteria informed specific management recommendations: reductions catch when *B*/*B*_0_ fell below *B_MSY_*/*B*_0_, and increased size at first capture when *L_c_* remained below *L_c_opt_*, thereby supporting stock recovery and sustainable yield [11].

## 3. Results

**Figure 2:**
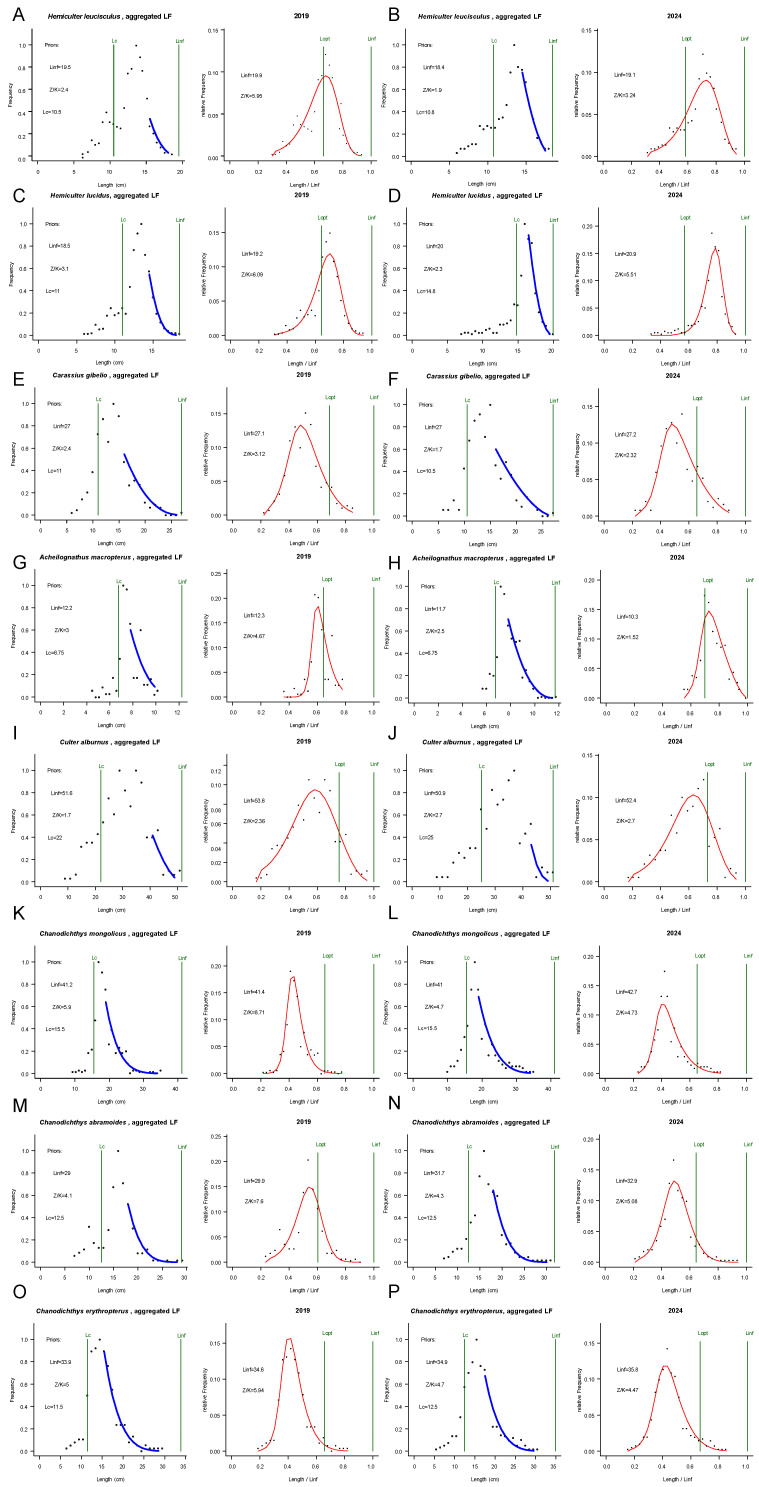
Length-frequency data and fitted LBB curves for eight fish species in Xingkai Lake, 2019 and 2024. For each species (**A**–**P**), the left panel illustrates the alignment between the model (blue lines) and the empirical length-frequency data, while the right panel displays the resultant stock status output (red lines) from the LBB analysis.

The LBB assessment of eight fish species in Xingkai Lake revealed significant changes in stock status between 2019 and 2024, following the initial implementation of uniform fishing gear regulations in 2019 and five years of subsequent management. The analysis was based on a total of 5485 individuals, comprising 2705 samples from the 2019 survey and 2780 from the 2024 survey. The findings are presented below by species, followed by a general synthesis. Key biological and prior parameters are summarized in Table 1, while stock assessment indicators and status classifications are presented in Table 2. Figure 2 displays the fitted LBB curves for the eight species in both 2019 and 2024, showing a good model fit to the length-frequency data across both assessment years.

### 3.1. H. leucisculus

For *H. leucisculus*, the *F*/*M* ratio declined from 2.87 to 0.52, indicating a reduction in fishing mortality below the natural mortality rate. The *B*/*B*_0_ ratio increased from 0.22 to 0.67, reaching approximately two-thirds of the unexploited level. The stock recovered from an overfished to a healthy status, with *B*/*B_MSY_* increasing from 0.59 to 1.90, indicating that current biomass exceeds the level required for maximum sustainable yield. The parameter *E* declined from 0.73 to 0.34, and *Y*/*R*′ decreased from 0.041 to 0.010, suggesting underutilization of the resource. The *L_c_*/*L_c_opt_* ratio increased from 1.2 to 1.7, indicating a shift in the population structure toward that of an unexploited stock.

### 3.2. H. lucidus

For *H. lucidus*, the reduction in fishing pressure was less pronounced than in its congener *H. leucisculus*. The *Z*/*K* ratio declined from 6.08 to 5.50, and the *F*/*M* ratio decreased from 2.69 to 1.40. The *B*/*B*_0_ and *B*/*B_MSY_* ratios increased from 0.23 to 0.42 and from 0.64 to 1.20, respectively, indicating a transition from an overfished to a healthy status. The *L_c_*/*L_c_opt_* ratio increased from 1.2 to 1.6, indicating a dominance of larger individuals in the catch, a pattern consistent with that observed in its congener, *H. leucisculus*.

### 3.3. Ca. gibelio

For *Ca. gibelio*, the *F*/*M* ratio declined from 1.27 to 0.48, indicating a reduction in fishing mortality below the natural mortality rate. Parameter *E* decreased from 0.57 to 0.32, suggesting a low level of resource utilization. The stock recovered from an overfished to a healthy status, with the *B*/*B*_0_ ratio increasing from 0.25 to 0.51 and the *B*/*B_MSY_* ratio rising from 0.67 to 1.40. Both the *L_mean_/L_opt_* and *L_95th_/L_inf_* ratios remained below 0.9, indicating a scarcity of larger individuals in the population.

### 3.4. A. macropterus

For *A. macropterus*, a substantial reduction in fishing pressure was observed. The *F*/*M* ratio decreased from 1.81 to 0.19, and parameter *E* declined from 0.65 to 0.16. The *B*/*B*_0_ ratio increased from 0.23 to 0.80, approaching the unexploited state. The stock recovered from an overfished to a healthy status, with *B*/*B_MSY_* increasing from 0.64 to 2.10. The *L_95th_/L_inf_* ratio increased from 0.78 to 0.99, indicating a high proportion of large individuals in the population.

### 3.5. Cu. alburnus

For *Cu. alburnus*, the *Z*/*K* ratio increased from 2.35 to 2.70 and the *F*/*M* ratio rose from 1.40 to 1.46, making it the only species where fishing pressure increased following the comprehensive implementation of gear regulations. The population maintained an overfished status despite increases in the *B*/*B*_0_ ratio (from 0.27 to 0.30) and the *B*/*B_MSY_* ratio (from 0.69 to 0.78). The *L_mean_/L_opt_* ratio increased from 0.91 to 0.99, indicating that the average size in the catch approached the optimal length. However, the *L_95th_/L_inf_* ratio decreased from 0.95 to 0.93, reflecting a decline in the proportion of the largest individuals.

### 3.6. Ch. mongolicus

For *Ch. mongolicus*, a marked decline in fishing pressure was observed. The *Z*/*K* ratio decreased from 8.74 to 4.75, and the *F*/*M* ratio from 4.44 to 1.98. The stock transitioned from a collapsed to a grossly overfished status, with the *B*/*B*_0_ ratio increasing from 0.06 to 0.14 and the *B*/*B_MSY_* ratio increasing from 0.17 to 0.40. Although the *L_95th_/L_inf_* ratio improved from 0.77 to 0.82, values below 0.9, indicating a continued scarcity of larger individuals.

### 3.7. Ch. abramoides

For *Ch. abramoides*, the *B*/*B*_0_ ratio increased marginally from 0.15 to 0.17, remaining below one-fifth of the unexploited level. The *Z*/*K* ratio declined from 7.65 to 5.07, with the parameter *E* from 0.74 to 0.68, indicating moderated fishing pressure. The stock maintained a grossly overfished status despite a slight rise in the *B*/*B_MSY_* ratio from 0.44 to 0.46. The *L_mean_/L_opt_* ratio declined from 1.0 to 0.85, indicating a trend of size truncation. The *L_c_*/*L_c_opt_* ratio decreased from 1.0 to 0.79, indicating a widening gap between the actual and optimal length at first capture.

### 3.8. Ch. erythropterus

For *Ch. erythropterus*, the *B*/*B*_0_ ratio improved from 0.09 to 0.14, though biomass remained severely depleted. The stock maintained a grossly overfished status, with the *B*/*B_MSY_* ratio increasing from 0.26 to 0.39. The *F*/*M* ratio decreased from 2.76 to 1.99. The *L_95th_/L_inf_* ratio increased from 0.82 to 0.85, indicating a greater presence of larger individuals. The *L_c_*/*L_c_opt_* ratio remained near 0.62, well below the optimal value.

Overall, the comparative LBB assessment between 2019 and 2024 revealed divergent trajectories in stock status among the eight species. Four species—*H. leucisculus*, *H. lucidus*, *Ca. gibelio*, and *A*. *macropterus*—recovered to healthy status (*B*/*B_MSY_* > 1) by 2024, while *Cu. alburnus* remained overfished and three species (*Ch. abramoides*, *Ch. mongolicus*, and *Ch. erythropterus*) maintained grossly overfished status with *B*/*B*_0_ values below 0.5. Except for *A. macropterus*, *H. lucidus* and *H. leucisculus*, all species showed *L_c_*/*L_c_opt_* ratios below unity, indicating suboptimal size at first capture. Similarly, three species—*Ca. gibelio*, *Ch. mongolicus*, and *Ch. erythropterus*—showed *L_95th_/L_inf_* values below 0.9, reflecting a limited presence of larger individuals in their populations. Notably, *Cu. alburnus* was the only species where fishing pressure increased despite management implementation.

## 4. Discussion

Scientific assessment of fishery exploitation status is essential for developing sound management strategies and ensuring sustainable utilization of fishery resources [25]. This study presents the first application of the Length-Based Bayesian Biomass (LBB) estimation method to assess the impact of a mesh-size increase policy, implemented five years prior, on the stock status of eight major fish species in Xingkai Lake.

### 4.1. Effectiveness and Limitations of the Uniform Mesh-Size Policy

The implementation of uniform mesh-size regulations may not achieve optimal outcomes simultaneously for all species, with the initial depletion level of a stock critically influencing its recovery trajectory [26,27]. Our results demonstrate that the larger mesh size regulation implemented between 2019 and 2024 effectively facilitated the recovery of most fish stocks, as indicated by multiple key assessment metrics. The most notable improvement was the substantial reduction in fishing pressure, with *F*/*M* ratios decreasing in seven of the eight assessed species. The reduction in fishing mortality facilitated biomass recovery, as all evaluated species exhibited a general upward trend in both *B*/*B*_0_ and *B*/*B_MSY_* values. Particularly, four species (*Ca. gibelio*, *A*. *macropterus*, *H*. *lucidus*, and *H. leucisculus*) transitioned to healthy status (*B*/*B_MSY_* > 1), indicating successful recovery to biomass levels capable of sustaining maximum sustainable yield. However, *Cu. alburnus* remained overfished, while three other species (*Ch. abramoides*, *Ch. mongolicus*, and *Ch. erythropterus*) remained in a grossly overfished status. These differential responses can be explained by the selective nature of gillnet fishing. As a highly size-selective fishing gear, gillnets primarily capture individuals within specific length ranges determined by mesh size [28]. The implementation of larger meshes created size-selective escape opportunities that particularly benefited smaller-bodied species, allowing a greater proportion of juveniles to reach reproductive maturity and consequently enhancing their recovery rates. However, this mesh size optimization proved less effective for medium-to-large piscivorous species such as those in the genus *Chanodichthys* and *Cu. alburnus* for two primary reasons. First, the selected mesh size likely remained suboptimal for achieving the target size at first capture. Second, their extremely low *B*/*B_MSY_* levels—particularly evident in *Ch. mongolicus*, which registered 0.17 in 2019—place these stocks in a collapsed state. This condition is associated with substantially extended and more variable recovery timelines, according to existing research [29]. While a decade may suffice for rebuilding overfished stocks (0.5 > *B*/*B_MSY_* ≥ 0.2), collapsed stocks (*B*/*B_MSY_* < 0.2) typically require considerably longer recovery periods [27].

### 4.2. Ecosystem Impacts and Community Restructuring

The implementation of uniform management measures, without considering complex species interactions within ecosystems, often fails to achieve expected conservation outcomes and may lead to unintended consequences [30]. The *B*/*B*_0_ ratio is a crucial indicator for evaluating fishery resource status, reflecting both exploitation intensity and population health, with values below 0.2 widely recognized as a critical threshold indicating stock collapse [11]. Our study revealed that although *Ch. abramoides*, *Ch. mongolicus*, *Ch. erythropterus*, and *Cu. alburnus* showed marginal improvements in their *B*/*B*_0_ ratios, these values remain critically low. Of particular concern, three of these species maintain biomass levels indicative of stock collapse (*B*/*B*_0_ < 0.2). Although fishing gear restrictions imposed since 2019 have contributed to reduced fishing pressure, medium and large-sized fish stocks remain severely depleted. This species-specific response highlights a potential ecological concern: the uniform management approach in Xingkai Lake may be promoting structural miniaturization of the fish community. This shift involves a transition from assemblages dominated by long-lived, high-trophic-level species toward communities characterized by short-lived, small-bodied, low-trophic-level organisms [31]. The successfully recovered species in this study are r-strategists, characterized by high growth rates, early maturation, and short lifespan [32]. The increased mesh size effectively reduced fishing pressure on these species, while their high intrinsic population growth rates enabled rapid responses to management measures, and resulted in swift biomass recovery. The population expansion of these small fish species intensifies interspecific competition for food and spatial resources, potentially further constraining the ecological niches of already vulnerable populations [33]. This shift may trigger cascading effects at the ecosystem level. As these small fish serve as important prey for piscivorous species but primarily consume zooplankton, their population increase could reduce zooplankton biomass and shift the community structure toward smaller species [34]. This trophic interaction weakens a critical constraint on phytoplankton growth. In potentially eutrophic water bodies like Xingkai Lake, this disruption of top-down control, driven by changes in fish population structure, may increase the risk of cyanobacterial blooms [35].

### 4.3. Drivers of Resource Decline

The slow recovery of fishery resources in Xingkai Lake resulted from the combined effects of historical overfishing, ecological degradation, and management deficiencies. Overfishing represented a principal causative factor. The fishing fleet expanded from 30 vessels in the 1960s to over 220 currently, while the number of triple-layer gillnets increased from approximately 3400 in 2001 to nearly 6000 today [17]. Parallel to this expansion, the annual catch surged from 492 tons in 2011 to approximately 2000 tons currently [17]. This intensive fishing effort, combined with the use of inappropriate mesh sizes prior to 2019, caused severe declines in stock abundance and recruitment. A representative example was *Cu. alburnus*, whose contribution to total catch plummeted from over 80% during the 1980s to merely 5% at present, reflecting a persistent declining trend [18]. Overfishing affected not only targeted species but also had cascading effects on others through habitat damage and disruption of ecological interactions [36]. The practice, prevalent before 2019, of annually harvesting over 10 tons of small adult fish for fishmeal constituted a clear case of growth overfishing, as individuals were captured before reaching their optimal size [17]. Concurrently, the widespread capture of immature individuals of larger species led to recruitment overfishing, severely undermining the reproductive capacity of key populations [17]. These combined pressures truncated the population age and size structure, fundamentally compromising resilience and long-term productive potential. Theoretically, optimal yield occurs when fishing mortality equals natural mortality (*F* = *M*), with an exploitation rate (*E*) of 0.5 often considered the reference point for optimal harvest [37]. This study demonstrated that *Ca. gibelio*, *A*. *macropterus*, and *H*. *leucisculus* showed clear signs of underexploitation (*E* < 0.5; *F*/*M* < 1.0), suggesting potential for increased sustainable utilization. However, most investigated fish stocks continued to experience fishing pressure exceeding their sustainable capacity. This persistent overfishing, compounded by emerging ecological interactions, underscores the severity of the situation. The successful recovery of small planktivorous fish, while a positive outcome for these species, may inadvertently trigger a trophic cascade. By suppressing zooplankton grazers, their population increase could elevate phytoplankton biomass, thereby heightening the risk of algal blooms in the eutrophic Xingkai Lake. This dynamic underscores that future management improvements must account for these complex species interactions.

Severe habitat degradation has significantly accelerated fishery resource decline over the past five decades, as evidenced by wetland area reduction at an average rate of 9004 hectares annually [38]. Wetlands provide crucial ecological functions including water conservation, purification, flood regulation, and biodiversity maintenance [39]. Rapid wetland shrinkage and fragmentation in the Xingkai Lake Basin have reduced critical spawning grounds for phytophilous fish species, changes primarily driven by socioeconomic development with additional influence from changing climatic conditions [38]. Concurrently, destruction of riparian vegetation along the northern shore has accelerated siltation of gravel-substrate lake beds, eliminating essential spawning habitats for species such as *Cu. alburnus* and *Ch. abramoides* [18]. As a key pressure operating independently of in-lake fisheries measures, this habitat loss presents a fundamental challenge to stock recovery. This habitat loss creates a critical recruitment bottleneck by directly compromising reproductive success. Furthermore, group-spawning fish naturally congregate in specific spawning grounds [40]. The spatial compression of these spawning grounds concentrates aggregating adults into more confined areas, thereby substantially increasing their vulnerability to capture. Therefore, any management strategy that focus fishing pressure on the remaining large, reproductively active individuals would be counterproductive, as it would further exacerbate recruitment overfishing. This interplay underscores the necessity of integrating habitat restoration with fisheries regulations that specifically protect spawners to effectively alleviate this bottleneck.

Environmental pollution presents an additional growing threat. Agricultural runoff has introduced increasing concentrations of organochlorine pesticides into the lake ecosystem, with frequent contamination incidents documented [17]. A study conducted in Xingkai Lake detected 37 different pesticides, with atrazine, simetryn, buprofezin, and isoprothiolane identified as predominant compounds; several substances including atrazine pose significant environmental risks to aquatic organisms [41]. The estimated annual nutrient load of 5.4 tons of total phosphorus and 7.6 tons of total nitrogen has driven the lake’s transition from oligotrophic to mesotrophic-eutrophic conditions [35]. Synergistic effects between eutrophication and toxic pesticide exposure create compounded ecological impacts, adversely affecting various physiological processes in fish including growth, reproduction, behavior, and swimming performance [42]. Notably, fish larvae at early developmental stages exhibit heightened vulnerability to both eutrophication-induced hypoxia and sublethal pesticide effects [43].

Current regulatory frameworks demonstrate insufficient control capacity. Fisheries management in Xingkai Lake relies primarily on fishing moratoria and mesh size restrictions but lacks effective control over total fishing mortality. Our findings reveal a paradoxical outcome of mesh size increases: fishing pressure intensified for valuable *Cu. alburnus*, while *L_c_*/*L_c_opt_* ratios declined for three *Chanodichthys* species. This suggests adaptive fisher behavior may offset conservation benefits through increased effort or targeting smaller individuals [44]. Management effectiveness is further complicated by socioeconomic pressures. In local economies with limited alternative livelihoods, fishing represents a critical income and food security source [19]. This economic vulnerability can lead to poaching and regulatory non-compliance [17]. To sustain livelihoods, some fishers resort to illegal activities, including off-season poaching or using destructive, prohibited fishing methods that yield higher short-term catches [45]. The persistent poverty-resource exploitation cycle proves difficult to break through conventional regulations alone, requiring nuanced approaches addressing both ecological conservation and human livelihood needs. The absence of comprehensive multi-species management strategies combined with limited enforcement capacity constitutes a fundamental constraint on the recovery of Xingkai Lake’s fishery resources.

### 4.4. Management Implications and Future Perspectives

Conventional fisheries management often prioritizes biomass increases in single or few target species, overlooking complex interspecific dynamics within ecosystems [30]. A more integrated approach combining multiple management measures would likely yield better outcomes [26]. Effective strategies for this wetland should link fisheries controls with habitat remediation. This includes enforcing spatially explicit fishing quotas, particularly for piscivorous species, coupled with habitat restoration programs targeting silted spawning beds and degraded riparian zones. Simultaneously, reducing external pressures by controlling nutrient and pesticide inputs from agricultural runoff is essential to create synergistic benefits across the ecosystem. Therefore, future management in Xingkai Lake should adopt an ecosystem-based adaptive framework. Current management comprises gear restrictions, size limits, and seasonal closures but lacks catch quotas. Management interventions should be prioritized based on specific stock-recovery objectives. For grossly overfished stocks such as *Ch. abramoides*, *Ch. mongolicus*, *and Ch. erythropterus*, the primary goal is spawning stock recovery, requiring the strictest protection through TACs or spatial closures at spawning sites. For overfished but recovering species like *Cu. alburnus*, measures should aim to reduce fishing mortality on juveniles and adults, combining the current mesh-size policy with effort controls. Finally, for healthy stocks, the objective shifts to optimizing yield within sustainable bounds. Adopting this graduated system of fishing controls ensures conservation resources are focused efficiently, reducing unnecessary burdens on stakeholders. Effective enforcement of catch limits necessitates robust monitoring of landings and catches to ensure accountability and traceability [46]. Extending the fishing moratorium from 6 June–15 July to 31 July is recommended to encompass the peak and late spawning periods of key commercial species, as supported by reproductive biology studies [17]. Concurrently, identifying and designating no-take zones requires prior mapping of critical spawning habitats through targeted surveys for submerged vegetation and gravel substrates. This evidence-based approach ensures that spatial protections are established in empirically defined locations, thereby safeguarding aggregations of spawning adults and juveniles with high certainty. Furthermore, stocking efforts for piscivorous species such as *Cu. alburnus* and *Ch. mongolicus* should be enhanced based on current ecosystem status to accelerate native predator recovery. This strategy supports trophic cascade effects that could help control small fish abundance and optimize community structure. For management to be effective, the scope of monitoring and assessment must match the scale of the ecosystem. We recommend prioritizing the establishment of a bilateral scientific and management framework for Xingkai Lake. The initial focus should be on harmonizing monitoring protocols and initiating regular data exchange between Chinese and Russian authorities. This will pave the way for developing coordinated management strategies, such as aligned fishing seasons and shared stock recovery objectives, which are essential to address the transboundary nature of the fish populations and avoid the pitfalls of fragmented, unilateral regulation.

## 5. Conclusions

This study assesses the fishery stock status in Xingkai Lake using the Length-Based Bayesian Biomass (LBB) method, evaluating the efficacy of uniform mesh-size regulations over a five-year period. The results reveal strongly species-specific recovery patterns and thereby demonstrate the inherent limitations of one-size-fits-all management measures in multi-species fisheries. The initial biomass level of a stock was identified as a critical factor determining its recovery trajectory. Consequently, the uniform gear regulation proved insufficient for ecosystem-wide recovery, particularly for severely depleted piscivorous species. Future management must adopt diagnostic, species-specific strategies tailored to the unique vulnerability of each stock. This research provides a scientifically grounded framework for reforming fisheries management in Xingkai Lake while offering a transferable template for assessing data-poor multi-species fisheries in freshwater ecosystems globally.

## Figures and Tables

**Table 1:** Basic information and prior parameters for eight fish species assessed using the LBB method in Xingkai Lake (2019 and 2024).

Scientist Name	Year	Min (cm)	Max (cm)	Class Interval	Numbers	*L_inf_* Prior (cm)	*Z*/*K* Prior	*M*/*K* Prior	*F*/*K* Prior	*L_c_* Prior (cm)	Alpha Prior
*H. leucisculus*	2019	5.5	18.5	0.5	396	19.5	2.4	1.5	0.94	10.7	12.5
	2024	5.5	18.0	0.5	444	18.4	1.9	1.5	0.36	11.0	14.7
*H. lucidus*	2019	5.5	18.5	0.5	631	18.5	3.1	1.5	1.56	11.2	16.4
	2024	6.5	20.0	0.5	439	20.0	2.3	1.5	0.75	15.0	35.3
*Ca. gibelio*	2019	5.0	27.0	1.0	292	27.0	2.4	1.5	0.87	11.2	17.9
	2024	5.0	27.0	1.0	251	27.0	1.7	1.5	0.16	10.7	21.0
*A. macropterus*	2019	4.2	10.2	0.3	169	12.2	3.0	1.5	1.46	6.9	48.0
	2024	5.4	11.7	0.3	347	11.7	2.5	1.5	0.96	6.9	39.7
*Cu. alburnus*	2019	8.0	51.0	2.0	266	51.6	1.7	1.5	0.22	22.4	8.4
	2024	8.0	51.0	2.0	192	50.9	2.7	1.5	1.19	25.5	8.9
*Ch. mongolicus*	2019	8.0	35.0	1.0	342	41.2	5.9	1.5	4.43	15.8	46.4
	2024	8.0	35.0	1.0	349	41.0	4.7	1.5	3.24	15.8	35.5
*Ch. abramoides*	2019	6.0	29.0	1.0	342	29.0	4.1	1.5	2.55	12.8	17.8
	2024	6.0	31.0	1.0	343	31.7	4.3	1.5	2.79	12.8	20.4
*C* *h* *. erythropterus*	2019	5.5	29.5	1.0	267	33.9	5.0	1.5	3.54	11.7	38.1
	2024	4.5	30.5	1.0	415	34.9	4.7	1.5	3.19	12.8	23.2

**Table 2:** LBB-based stock assessment estimates for eight fish species in Xingkai Lake (2019 and 2024).

Scientist Name	Year	*B*/*B*_0_	*B*/*B_msy_*	*F*/*M*	*Z*/*K*	*Y*/*R*′	*E*	*L_inf_* (cm)	*L_c_*/*L_c_opt_*	*L_mean_*/*L_opt_*	*L_95th_*/*L_inf_*	Stock Status
*H. leucisculus*	2019	0.22	0.59	2.87	6.03	0.041	0.73	19.9	1.20	1.20	0.93	overfished
	2024	0.67	1.90	0.52	3.22	0.010	0.34	19.1	1.70	1.40	0.94	healthy
*H. lucidus*	2019	0.23	0.64	2.69	6.08	0.037	0.73	19.2	1.20	1.20	0.94	overfished
	2024	0.42	1.20	1.40	5.50	0.011	0.59	20.9	1.60	1.50	0.93	healthy
*Ca. gibelio*	2019	0.25	0.67	1.27	3.09	0.050	0.57	27.1	0.71	0.81	0.85	overfished
	2024	0.51	1.40	0.48	2.34	0.033	0.32	27.2	0.79	0.84	0.88	healthy
*A. macropterus*	2019	0.23	0.64	1.81	4.67	0.041	0.65	12.3	0.99	0.99	0.78	overfished
	2024	0.80	2.10	0.19	1.55	0.020	0.16	10.3	1.30	1.10	0.99	healthy
*Cu. alburnus*	2019	0.27	0.69	1.40	2.35	0.084	0.59	53.6	0.84	0.91	0.95	overfished
	2024	0.30	0.78	1.46	2.70	0.072	0.59	52.4	0.99	0.99	0.93	overfished
*Ch. mongolicus*	2019	0.06	0.17	4.44	8.74	0.009	0.82	41.4	0.64	0.73	0.77	collapsed
	2024	0.14	0.40	1.98	4.75	0.022	0.66	42.7	0.62	0.73	0.82	grossly overfished
*Ch. abramoides*	2019	0.15	0.44	2.85	7.65	0.020	0.74	29.9	1.00	1.00	0.90	grossly overfished
	2024	0.17	0.46	2.06	5.07	0.027	0.68	32.9	0.79	0.85	0.94	grossly overfished
*C* *h* *. erythropterus*	2019	0.09	0.26	2.76	5.90	0.014	0.75	34.6	0.60	0.70	0.82	grossly overfished
	2024	0.14	0.39	1.99	4.46	0.023	0.67	35.8	0.62	0.73	0.85	grossly overfished

## Data Availability

All data are contained in the manuscript.
